# Did They Deserve It? Adolescents’ Perception of Online Harassment in a Real-Case Scenario

**DOI:** 10.3390/ijerph192417040

**Published:** 2022-12-19

**Authors:** Clarissa Cricenti, Alessandra Pizzo, Alessandro Quaglieri, Emanuela Mari, Pierluigi Cordellieri, Cristina Bonucchi, Patrizia Torretta, Anna Maria Giannini, Giulia Lausi

**Affiliations:** 1 Department of Psychology, Sapienza University of Rome, 00185 Rome, Italy; 2 State Police Postal and Communication Department, Ministry of the Interior, 00173 Rome, Italy

**Keywords:** cyber-crime, cyberbullying, victimization, social networking, moral disengagement, police force

## Abstract

Online harassment, particularly cyberbullying and the non-consensual sharing of intimate images, is a widespread phenomenon among adolescents and young adults. Descriptive research was carried out to investigate any differences among Italian school classes in the perception of cybercrime through a real-case scenario. Following the Italian school system, the final sample of 1777 adolescents (Mage = 15.37, SD = 1.65; Male = 52%) was divided into three groups based on the school class attended: middle school (*N* = 562; Mage = 13.37, SD = 0.48); high school biennium (*N* = 728; Mage = 15.55, SD = 0.50), and triennium (*N* = 487, Mage = 17.40, SD = 0.71). Participants completed a self-report questionnaire investigating the use of the Internet and the perception of a real case scenario involving the non-consensual sharing of intimate images and cyberbullying received by the National Centre for Combating Child Pornography Online (NCPO). Results showed differences among the three groups’ perceptions of the event’s features, motivations underlying the offense, victim-blaming and harassment justification (e.g., cyberbullying, in particular non-consensual sharing of intimate images, is recognized as a crime as age increases). The findings provide significant insights for future research and age-specific factors to consider when developing prevention programs for online risks.

## 1. Introduction

Since the development of the Internet, there has been a decrease of traditional offenses, especially in highly industrialized Western societies, which seems to be related to the evolution of cybercrime [1]. Cybercrime can be defined as “any crime (traditional or new) that can be conducted or enabled through, or using, digital technologies” [2], and can be conceptually divided into cyber-dependent crime (i.e., a crime that cannot be perpetrated without the internet, like hacking or spamming) and cyber-enabled crime (i.e., a traditional crime perpetrated in cyberspace to amplify its magnitude or reach using the internet, like online harassment) [1,3,4,5].

Online harassment, a cyber-enabled crime, refers to behaviors aimed at annoying, abusing, and tormenting people in cyberspace [6]. Online harassment appears to be composed of unique features [7]: widespread reach (i.e., the content is watched by many individuals), the permanence of the internet (i.e., the content is more difficult to remove, affecting victims at any time), and anonymity (i.e., the bullies are often unknown to victims) [4,8,9,10,11,12,13]. These features are linked to several adverse consequences for victims at the psychological (e.g., powerlessness, shame, fear, anxiety, depression symptoms), social, and economic (e.g., disruption of relationships and loss of work) levels [14,15]. Online harassment includes cyberbullying and the non-consensual sharing of intimate images. Cyberbullying can be defined as “an aggressive act or behavior that is carried out using electronic means by a group or an individual repeatedly and overtime against a victim who cannot easily defend him or herself” [16], while non-consensual sharing of intimate images is a new form of sexual abuse, also noted as a “Technology-Facilitated Sexual Violence” [17], defined by the non-consensual sharing of intimate and sexual visual content (i.e., images/videos) that can be obtained consensually or non-consensually [4,17,18,19]. This content is often shared along with the victim’s details (e.g., name, address), making them more vulnerable to abuse, stalking, and other forms of sexual harassment [15,20]. The non-consensual sharing of intimate images is used both by ex-partners to shame, extort, and harm victims as revenge following a break-up, by peers, family members, and co-workers [21].

Among the causes of online and offline aggressive behaviors, moral disengagement has been identified as a key factor [22,23,24,25,26] and is defined as the process by which individuals separate their moral norms from their immoral behaviors to avoid self-evaluation. It consists of four behavioral loci by which individuals regulate their conduct, including: justifying the behavior, shifting responsibility, minimizing the harm caused, and shifting the causal focus to the victim [27]. Moral disengagement predicts both sexual harassment and cyber-aggression by using: moral justification or diffusing responsibility to consider oneself as less responsible for one’s actions; euphemistic labeling, for example, considering these behaviors as funny or jokey; victim-blaming by attributing the responsibility to the victim, especially if the victims had sent the intimate images/videos consensually [25,28,29,30,31,32,33,34]. With regard to moral justification, few studies have analyzed the specific mechanism of moral disengagement. Thornberg and Jungert [35], found that moral justification was positively associated with bullying behavior in a sample of early adolescents (aged 10–14 years). Generally, the use of moral disengagement decreases in the developmental period from early adolescence (i.e., middle school) to adolescence (i.e., high school) [36,37,38]; in some cases, adolescents aged 14–15 years reported higher use of moral disengagement mechanisms than preadolescents (10–13-year-olds) [37,39]. Moreover, moral disengagement explains patterns of justification for the use of violence: children and adolescents who believe that it is appropriate to attack others when they deserve it are more likely to be aggressive [40,41,42,43]. Perren et al. [44] examined moral justification in adolescents aged 12–18 years as a function of self-reported bullying and victimization. Using a relational aggression vignette, teenagers were asked to explain the perpetrator’s perspective. Although there were no significant differences in the use of moral justification between pure bullies and bullies/victims, pure bullies had higher mean scores for these mechanisms than did bullying victims, which is consistent with a greater propensity toward violence in teens with high moral justification.

Interestingly, several studies on young victims of cyberbullying [45,46,47,48,49] have also found that adolescents tend to use moral justification as a way to empathize with their aggressors to protect their self-esteem, which could occur in victims of non-consensual intimate image sharing. Cyberbullying and non-consensual sharing of intimate images occur more among adolescents and young adults than older adults, often with females as victims [45,50,51,52,53,54], but non-consensual sharing of intimate images might be more evenly distributed among genders [55,56]. However, cyberbullying rates are related with the attended school class: as children move from primary school to middle and high school, the perpetration of cyberbullying decreases along with an increase in the ability to exert self-control [57,58,59]. Data showed a higher frequency of cyberbullying victims among 14–15 rather than 15–18-year-olds [60]. Moreover, cybercrime seems to differ according to age: adolescents reported higher editing of images/videos shared online; 14–15-year-olds are more likely to be victims of threats or insults; 12–13-year-olds are more likely to be victims of online rumor-spreading [61].

Such behaviors often occur largely in school, the primary place of socialization, where adolescents spend most of their time. The role of schools in cyberbullying and the non-consensual sharing of intimate images has, thus, been widely investigated. For example, school connectedness was found to moderate the relationship between cybervictimization and suicide risk in adolescents [62], while other studies [63] have shown that school educators often implicitly tolerate negative and non-inclusive attitudes, thus supporting the power structures that exist in a discriminatory school environment. In fact, in a study by Bevilacqua et al. [64], schools that performed well in terms of leadership and management generated protective school climates toward bullying and cyberbullying. Moreover, students in schools with voluntary assistance (e.g., religious schools) were less likely to be victims of such violent behavior than those in traditional state schools, which supports the idea of school ethics and culture as protective factors against cyberbullying [65].

Although the non-consensual sharing of intimate images and cyberbullying have been recognized as an offense in several countries [32,66,67], many people may not be aware that it is a crime, but rather would identify it as ingenious behavior or a funny joke [17]. Adolescents and young adults believe themselves to be aware of both the gravity and cybercrime of cyberbullying [68,69,70]. Indeed, an Australian study found that 99% of adolescents rated cyberbullying as “wrong” [71], and even pre-adolescents conceptualized bullying as morally transgressive because of the harm caused [72].

In addition, many adolescents believe that sexual images/videos remain private and are not shared on the internet [68,73,74,75], but there is a lack of knowledge about how cybercrime patterns change as age increases in adolescents [76,77]. In this regard, a study by Zilka [78] showed that the level of awareness about social media sharing among adolescents was medium-high and, specifically, it was lower in girls and older adolescents, as they share more content online, thus feeling more vulnerable and exposed than younger children.

### Aims

To the best of our knowledge, no previous studies have explored the perception of cyberbullying and the non-consensual sharing of intimate images as cybercrime in different attended school classes. As suggested by Bae [79], the greater the perception of cyberbullying as harmful and illegal, the more likely it is that the perpetration of cyberbullying will decrease.

Through a real case scenario, received by the National Centre for Combating Child Pornography Online (NCPO), the present study aimed to investigate the presence of any age difference in the evaluation of the event’s features, the motivations underlying the offense, the victim-blaming, and the justification of the subsequent harassment.

## 2. Materials and Methods

### 2.1. Participants

A total sample of 1874 participants from different schools in Italy were recruited. Inclusion criteria included attending middle or high school; speaking Italian; aging from 13 to 19 years old. Based on these criteria, the final sample consists of 1777 participants, 52% identifying as male (*N* = 940; age M = 15.37; SD = 1.65). According to the hypothesis of our study, the sample was divided into three groups based on the school class attended. In Italy, for adolescents, schools follows these steps: middle school (11–13 years old), which corresponds to years 7 to 9 of the UK system or to middle school in the USA (Grade 6 to 8), and high school, which lasts for 5 years and is divided in biennium (14–15 years old, corresponding to year 10-h11 of the UK system or Grade 9–10 of the USA system) and triennium (16–18 years old, corresponding to year 12–13 of UK system or Grade 11–12 of the USA system). Therefore, high school in the Italian school system lasts for five years instead of four, as in the other international systems.

Therefore, our sample was therefore divided into: middle school (*N* = 562; mean age = 13.37; SD = 0.48; 53.4% male); biennium, first two-year period of high school (*N* = 728; mean age = 15.55; SD = 0.50; 56.5% male); triennium, last three-year period of high school (*N* = 487, mean age = 17.40; SD = 0.71; 47% male). The demographic characteristics of the three groups are detailed in Table 1.

### 2.2. Procedures

Data were collected using the pen-and-paper procedure proposed by the Postal Police in Italian middle and high schools. All students agreed to participate, and their caregivers signed an informed consent form in which they were informed about the purpose of the study and the anonymity. Afterward, demographic characteristics, use of the Internet and social networks, and the perceptions of cybercrime were investigated with an ad hoc scenario and items. This study was conducted according to the ethical standards of the Helsinki Declaration and was approved by the Institutional Review Board of the Department of Psychology of “Sapienza” University of Rome (protocol number 0002195).

### 2.3. Materials

Demographic characteristics, such as sex, age, educational institution, and attended grade were collected. In addition, participants were asked to complete an ad hoc questionnaire consisting of questions about the use of the Internet and social networks and the perception of cyberbullying and the non-consensual sharing of intimate images committed by adolescents through an ad hoc scenario developed based on cases received by the National Centre for Combating Child Pornography Online (NCPO) (Table S1).

#### 2.3.1. Use of Internet

The use of the Internet and social networks was measured with 6 ad hoc items: 4 multiple-choice items assessed the most shared content and motivations for using social networks; 2 items, based on a 6-point Likert scale ranging from 1 (“not at all”) to 6 (“very much”), evaluated the diffusion of shared content (i.e., “In your opinion, how widely shared do you think the materials you post are?”) and daily use of social networks (i.e., “Approximately how much do you use social networks in a day?”).

#### 2.3.2. Perception of Cybercrime

The perception of cybercrime was measured using a real case scenario that refers to an episode of the diffusion of an intimate video involving a minor, where the perpetrators become, in turn, victims:

*“Fabio and Edoardo, both 16 years old, are deemed responsible for destroying Jessica’s reputation by spreading a consensual sexual video between Jessica and Edoardo. Francesco (16 years old) and Ludovica (17 years old) take action to defend Jessica, by insulting them, creating photomontages with heavy sexual allusions against them, threatening them with death, and intimidating them on social networks.”*

15 ad hoc items, based on a 6-point Likert scale ranging from 1 (“not at all”) to 6 (“very much”), based on Bandura’s moral disengagement theory (Table S2), were used to measure different aspects of the scenario: 4 items were related to event’s features (e.g., “Could this ever happen in the area where you are living?”); 4 items were related to motivations underlying the offense (e.g., “Do you think the authors planned for the consequences of their actions?”), these items can be related to the minimization or ignoring of the harm caused by the non-consensual sharing of intimate images; 3 items were related to victim-blaming (e.g., “Do you think Jessica may have violated any laws?”), defined by shifting the causal focus to the victims; 4 items were related to the justification of the harassment by the victim’s friends (e.g., “Do you think the reaction against Fabio and Edoardo is understandable?”), related to the belief that cyberbullying behaviors in defense of a victim are justifiable, shifting responsibility for one’s actions.

### 2.4. Data Analysis

Data Statistical analyses were conducted using SPSS (Statistical Package for Social Science; version 27.0; IMB SPSS; Armonk, NY, USA). First, descriptive analyses of sample characteristics and use of the Internet and social networks were performed. Then, the data distributions were verified for normality: two items (i.e., “Could you ever do what Fabio and Edoardo did?” and “could you ever do what Jessica did?”) showed high values for symmetry (=2.47 and =2.03, respectively); after applying the reciprocal transformation, all variables lower than 2.0 for skewness and 7.0 for kurtosis were corrected; therefore, the distribution was considered normal (Curran et al., 1996). A Chi-Square Test with post-hoc Z-test for independent proportions was used to compare the use of social networks and the perception of the dissemination of shared material between age groups. Finally, through analysis of variance (ANOVA), differences between groups on the perception of cybercrime were investigated. Statistical significance in the post-hoc analysis was determined using Bonferroni correction and defined as *p* < 0.05.

## 3. Results

### 3.1. Descriptive Analysis on Use of Internet

Frequency analysis showed that social networks are mainly used for socializing and the most frequent types of shared content are photos and messages, through smartphones, in all students’ groups (Table 2). However, among the three groups, the motivations of “curiosity” showed significantly higher frequency in biennium and triennium students, “flirting” showed significantly higher frequency in triennium students, and “finding information” showed significantly higher frequency in middle and triennium students. Similarly, significant differences emerged in the content shared on social networks, where triennium students would send more photos and tweets, while posts were sent with significantly higher frequency by middle and triennium students. Facebook and Instagram are the most-used social networks for all student groups. However, Facebook use is more frequent among middle and triennium students, while Instagram use is more frequent among biennium and triennium students.

Concerning the accessibility of shared materials, most of the students in the biennium and triennium groups believed that everyone has access to their content, while in the middle school group, there is a higher percentage of students who believe that the shared materials are accessible only to the recipient. Regarding statistically significant differences between the groups, the belief that the materials are accessible only to the recipient is more frequent among middle school and biennium students, while the belief that the materials are accessible to one’s network is more frequent among triennium students than among the other groups.

Finally, all groups of students report moderate Internet use and perceive moderate spread of the online materials they share. Differences between the groups show that middle school students use the Internet significantly less than biennium and triennium students.

### 3.2. Differences between Groups on Perception of Cybercrime

**Figure 1:**
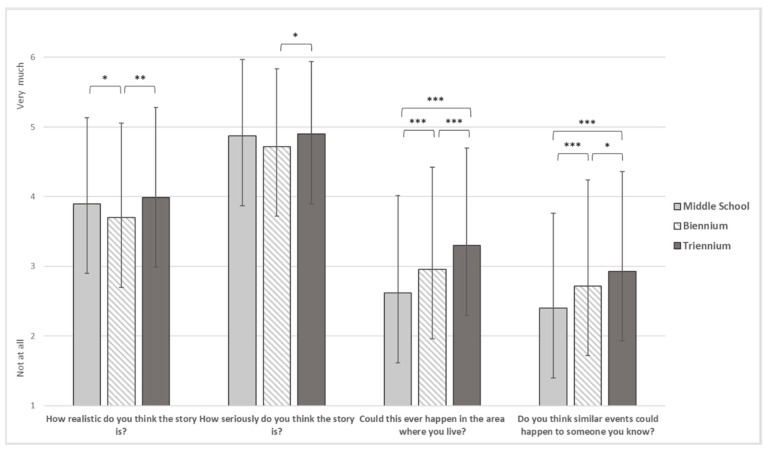
Event’s Features. Note. * = *p* < 0.05, ** = *p* < 0.01, *** = *p* < 0.001.

With regard to the item investigating the perception of cybercrime, statistically significant results emerged in all items concerning the event’s features (Figure 1). Biennium students reported a significantly lower mean (M = 3.70, SD = 1.353) than both triennium (M = 3.99, SD = 1.288) and middle students (M = 3.90, SD = 1.232) regarding the credibility of the scenario. Moreover, triennium students reported significantly higher mean (M = 4.90, SD = 1.039) than biennium students (M = 4.72, SD = 1.117) on the perception of the severity of the event. Finally, concerning both the event’s physical proximity and the possibility that acquaintances may experience the event, the results showed a significantly higher mean among triennium students (M = 3.30, SD = 1.398 and M = 2.93, SD = 1.429, respectively) than both middle (M = 2.62, SD = 1.399 and M = 2.40, SD = 1.366) and biennium students (M = 2.96, SD = 1.463 and M = 2.72, SD = 1.521), and biennium students reported a significantly higher mean than middle students.

**Figure 2:**
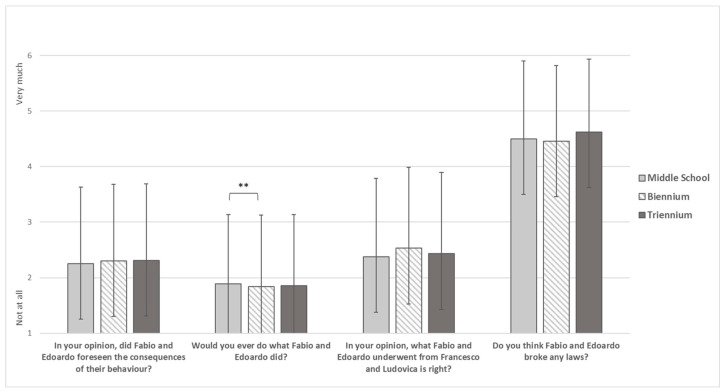
Motivations underlying the offense Note. ** = *p* < 0.01.

With regard to the items on motivations underlying the commission of the non-consensual sharing of intimate images offense (Figure 2), there were no statistically significant results regarding the foresight of the consequences of their actions by offenders (F_(2)_ = 0.275, *p* = 0.760), offenders law violation (F_(2)_ = 2.183, *p* = 0.113), and victim-blaming (F_(2)_ = 1.848, *p* = 0.158). By contrast, middle students reported a significantly higher mean (M = 0.89, SD = 0.24) than biennium students (M = 0.84, SD = 0.29) on the likelihood of behaving like offenders.

**Figure 3:**
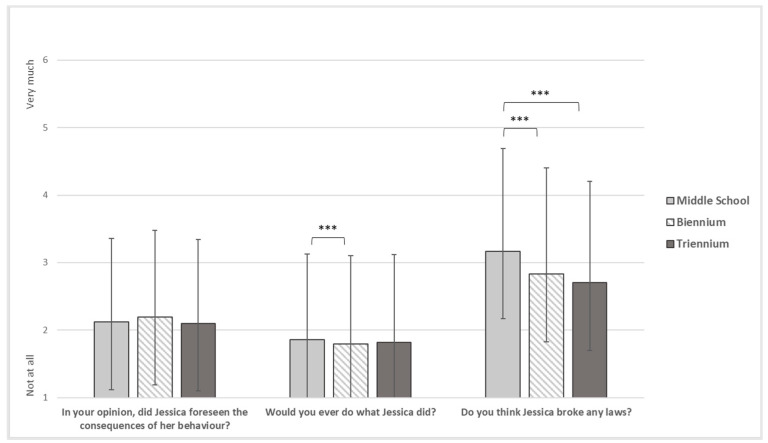
Victim-blaming. Note. *** = *p* < 0.001.

As for the victim-blaming items (Figure 3), there were no statistically significant results regarding the foresight of the consequences of her actions by the victim (F_(2)_ = 0.786, *p* = 0.456). By contrast, middle students reported a significantly higher mean (M = 0.86, SD = 0.27) than biennium students (M = 0.79, SD = 0.31) on the likelihood of behaving like the victim. Significant differences also emerged regarding the violation of the law by the victim, with significantly higher means for middle students (M = 3.17, SD = 1.520) than for both biennium (M = 2.83, SD = 1.58) and triennium students (M = 2.70, SD = 1.507).

**Figure 4:**
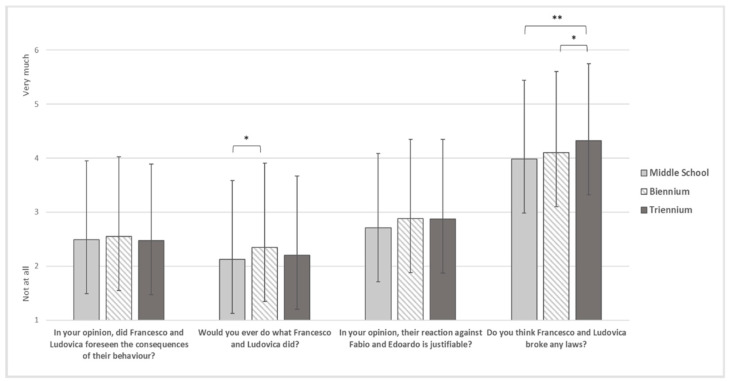
Justification of the harassment by the victim’s friends. Note. * = *p* < 0.05, ** = *p* < 0.01.

Finally, with regard to the justification of the harassment by the victim’s friends (Figure 4), there were no statistically significant results regarding the foresight of the consequences of their actions by offenders (F_(2)_ = 0.470, *p* = 0.625), and justification of their reaction (F_(2)_ = 2.687, *p* = 0.068). However, biennium students showed significantly higher means (M = 2.35, SD = 1.553) than middle students (M = 2.13, SD = 1.455) on the likelihood of behaving like the offenders, and triennium students (M = 4.32, SD = 1.428) reported significantly higher means than both biennium (M = 4.10, SD = 1.505) and middle students (M = 3.98, SD = 1.460) on violation of the law by offenders.

## 4. Discussion

The study aimed to investigate the perception of cybercrimes, specifically, the non-consensual sharing of intimate images and cyberbullying in different grades of secondary schools (i.e., middle school, high school biennium, and triennium).

The results showed that triennium students perceived the scenario as more serious and credible than did biennium students. Although the prevalence of cyberbullying decreases between 13 and 18 years old [57,58,59,60], as also shown by the frequency analysis on Internet use within the present study, the spreading and editing of images/videos shared online increased with age [61], with a probable intensification in the awareness of this crime. Interestingly, middle school students perceived greater trustworthiness of the scenario than did biennium students. During the early years of high school, adolescents begin to move away from the family context and develop important friendships and intimate relationships involved in the development of moral reasoning [80]. Therefore, the events described in the scenario could be evaluated as morally unacceptable within their social context, and unrealistic. In addition, the biennium students could identify with the characters of the scenario, being their peers, while the middle students could experience the scenario as more distant from their own, and yet no less real. Although middle school students considered the story more plausible than did biennium students, the perceived closeness of the event increased with age, consistent with the development of affectively and sexually connoted romantic relationships [81] and with increased involvement in sexting [82,83].

Moreover, younger people are more overconfident, although they are more impulsive and more able to avoid risks [84,85,86]. Younger adolescents engage in dangerous activities even when they know and understand the risks involved, but their actions are mainly guided by feelings and social influences [87,88,89]. Regardless of the attended school class, nonconsensual sharing of intimate images was perceived as a violation of the law; however, only middle school students would engage more in the non-consensual sharing of intimate images and victim behavior than biennium students. This could be related to the higher frequency in middle school students of the belief that shared material is only accessible to the recipient. For cyberbullying, the opposite pattern is observed, along with an increase in law violation recognition as age increases. These results could be related to the higher prevalence of cyberbullying (i.e., hate crimes) in middle school students [61] and, therefore, higher novelty-seeking and the underestimation of risks and overconfidence. Moreover, as we grow up, metacognitive skills and internalization of moral principles improve and, with them, the self-regulation skills related to self-evaluation mechanisms [37,90,91,92,93,94]. Therefore, older adolescents may engage less in the non-consensual sharing of intimate images but may use cyberbullying to a greater extent as a form of revenge, a response related to the use of moral disengagement mechanisms, in particular, the moral justification mechanism used to redefine the meaning of the action as being by socially accepted principles, such as honor. Both the revenge response and disengagement mechanisms may be more common among biennium students than both middle and triennium students [36,37,38,95,96].

Finally, the results showed that the victims’ perception of violation of the law was higher among middle students than both the biennium and triennium students, consistent with greater use among younger adolescents of moral disengagement mechanisms, such as victim-blaming, resulting in greater feelings of responsibility by the victim for what happened [25,28,29,30,31,32,33,34].

Since schools are places where children and adolescents first socialize and educate themselves, as well as develop online and offline relationships, moral behavior, and communication, there is a wide range of school-based interventions aimed at preventing online sexual violence, including the sharing of unwanted intimate sexual messages [97]. However, many of these programs merely focus on abstinence from sexting and the use of risk communication strategies to discourage sexting altogether, while no alternative digital sexual education interventions have been observed [98,99]. However, previous studies have shown that there is a need to develop prevention programs that empower students in the face of cyberbullying and intimidation on social media and in other online environments [100,101,102,103]. In addition, programs focused on the effects on both victims and perpetrators can have a series of positive effects on both the school environment and adolescents [104]. Younger students (i.e., those in middle school) may benefit from a specific intervention aimed at learning more about age-related risk factors and the sharing of intimate images, as several studies have revealed different patterns of early onset sexting compared to sexting in later adolescence [105,106]. Early prevention programs focused on developing targeted communication (e.g., assertiveness) and self-regulation skills for this specific target could prove useful in improving gradual empowerment to deal with the risks of cyberbullying and the sharing of intimate images online. Hence, Manzuoli and Medina [107] argued that early adolescents in cyberbullying situations could be better prepared to deal with this threat through response education that includes actions such as seeking support from adults (e.g., parents, relatives, or teachers) and/or government organizations; hiding, deleting, and/or deactivating social media account features in order to eliminate or reduce unsolicited/unwanted communications; and being communicatively assertive and making effective and timely decisions. Conversely, older adolescents (i.e., biennium and triennium students), who usually share more intimate content online, may benefit from peer-educational school programs on sexting and intimate image sharing, achieving encouraging outcomes in terms of knowledge acquisition with respect to the possible risks and consequences of such behavior, with a greater effectiveness of peer-to-peer communication in spite of institutional intervention, which is often based on abstinence and seems to be, in some way, judgmental [108].

In any case, identification of the presence of cyberbullying and examination of the possible correlations among the types and factors that influence school violence are necessary steps for comprehensive needs analysis to help educational agents and stakeholders better understand their educational communities and, thus, develop more effective cyberbullying prevention plans and long-lasting protective environments for adolescents and their families.

## 5. Conclusions

The study presented is descriptive research, designed to investigate possible differences between age groups in the perception of cybercrime. The scenario involved two crimes (i.e., the non-consensual sharing of intimate images and cyberbullying) and two forms of victimization, implying the use of different moral judgments in attributing realism and severity to the scenario. Future studies could use the scenario to assess how moral disengagement mechanisms act in evaluating realism and blaming attribution by breaking down the scenario according to the desired aspects to be emphasized. Notably, this study can already act as a groundwork for the development of both online safety and digital communication education programs, so that they can be designed to act differently for specific age groups, depending on what is relevant to each one. Moreover, this study could be repeated in an adult population (e.g., parents, caregivers), to understand their awareness of how children communicate and the risks of the network.

This study, however, has some limitations: on the one hand, being a true story is a strength for reliability and realism; but, on the other hand, it places a limit on the replicability of the scenario. Indeed, it is difficult to find more than one scenario with the same story but differing in gender and age of the victim and author. Furthermore, while this study is descriptive (and therefore does not investigate psychological mechanisms, but rather gives an overview of these two cybercrimes), the lack of validated scales, specifically about moral disengagement, is a limitation that was not part of the research objectives. Hence, since the questionnaire was administered by police forces, the data may be subjective to social desirability bias; however, the ethical implications were considered in dealing with data through the training of the police force. Moreover, before each administration, the research project was explained to teacher in order to better introduce the police officials to the class, further reducing the bias.

## Figures and Tables

**Table 1:** Demographic characteristic.

		Middle School	Biennium	Triennium
Age	Mean	13.37	15.55	17.40
	Std. Deviation	0.48	0.50	0.71
Gender	Female	262 (46.6%)	317 (43.5%)	258 (53.0%)
	Male	300 (53.4%)	411 (56.5%)	229(47.0%)
	Total	562 (100%)	728 (100%)	487 (100%)

**Table 2:** Frequency Analysis of use of the Internet in student groups.

		Middle School		Biennium		Triennium		Chi-Square		
		*N*	%	*N*	%	*N*	%	X^2^	df	*p*
Why do you use these social networks?	Socializing	366 ^a^	65.1	459 ^a^	63.3	299 ^a^	61.9	1.182	2	0.554
	Curiosity	261 ^a^	46.4	411 ^b^	56.7	287 ^b^	59.4	20.740	2	0.000
	Show more sides of me	29 ^a^	5.2	39 ^a^	5.4	32 ^a^	6.6	1.214	2	0.545
	Flirting	27 ^a^	4.8	69 ^b^	9.5	68 ^c^	14.1	26.669	2	0.000
	Find Information	198 ^a^	35.2	206 ^b^	28.4	179 ^a^	37.1	11.772	2	0.003
What kind of material do you share most?	Photos	319 ^a^	56.8	415 ^a^	57.2	339 ^b^	69.9	24.438	2	0.000
	Videos	130 ^a^	23.1	174 ^a^	24.0	98 ^a^	20.2	2.478	2	0.290
	Messages	376 ^a^	66.9	434 ^b^	59.9	301 ^a,b^	62.1	6.828	2	0.033
	Tweets	55 ^a^	9.8	73 ^a^	10.1	77 ^b^	15.9	12.135	2	0.002
	News	101 ^a^	18.0	123 ^a^	17.0	74 ^a^	15.3	1.390	2	0.499
	Others	32 ^a^	5.7	50 ^a^	6.9	34 ^a^	7.0	0.983	2	0.612
Which social networks do you use the most?	Instagram	270 ^a,b^	48.0	331 ^b^	45.5	264 ^a^	54.3	9.167	2	0.01
	Facebook	282 ^a^	50.2	439 ^b^	60.4	321 ^b^	66.0	28.516	2	0.000
	WhatsApp	506 ^a^	90.0	639 ^a^	87.9	436 ^a^	89.7	1.774	2	0.412
	Twitter	34 ^a^	6.0	39 ^a^	5.4	31 ^a^	6.4	0.597	2	0.742
	Other	110 ^a^	19.6	166 ^a^	22.8	104 ^a^	21.4	3.050	4	0.549
Through which devices?	Smartphone	535 ^a^	95.2	676 ^a^	93.0	464 ^a^	95.5	4.457	2	0.108
	Shared Laptop	18 ^a^	3.2	35 ^a^	4.8	30 ^a^	6.2	5.210	2	0.074
	Personal Laptop	98 ^a^	17.4	115 ^a^	15.8	126 ^b^	25.9	20.727	2	0.000
	Tablet	104 ^a^	18.5	128 ^a^	17.6	76 ^a^	15.6	1.550	2	0.461
	Others	13 ^a^	2.3	24 ^a^	3.3	13 ^a^	2.7	1.180	2	0.554
Who do you think the material you share is accessible to?	Everyone	176 ^a^	34.4	245 ^a^	35.7	161 ^a^	35.2	33.607	10	0.000
	Recipient	195 ^a^	38.2	223 ^a^	32.5	114 ^b^	24.9			
	My network only	113 ^a^	22.1	173 ^a^	25.2	157 ^b^	34.3			
	Adults	5 ^a^	1.0	8 ^a^	1.2	3 ^a^	0.7			
	Other	19 ^a^	3.7	24 ^a^	3.5	19 ^a^	4.1			
How much do you use social networks in a day?	Never	15 ^a^	2.7	16 ^a^	2.2	7 ^a^	1.4	49.878	10	0.000
	Almost Never	46 ^a^	8.2	32 ^b^	4.4	14 ^b^	2.9			
	Rarely	121 ^a^	21.7	124 ^a,b^	17.1	61 ^b^	12.6			
	Sometimes	217 ^a^	38.9	328 ^a^	45.2	200 ^a^	41.2			
	Almost Always	128 ^a^	22.9	181 ^a^	25.0	157 ^b^	32.4			
	Always	31 ^a^	5.6	44 ^a,b^	6.1	46 ^b^	9.5			
How widespread do you think the material you share is?	Not at all spread	66 ^a^	12.0	79 ^a,b^	11.1	34 ^b^	7.2	10.483	10	0.399
	Low spread	64 ^a^	11.6	85 ^a^	12.0	61 ^a^	12.8			
	Slightly spread	167 ^a^	30.4	207 ^a^	29.1	131 ^a^	27.6			
	Moderately spread	180 ^a^	32.7	241 ^a^	33.9	176 ^a^	37.1			
	Very spread	57 ^a^	10.4	72 ^a^	10.1	54 ^a^	11.4			
	Extremely spread	16 ^a^	2.9	27 ^a^	3.8	19 ^a^	4.0			

Note. Each superscript letter indicates which differences are significant and which are not significant at the specified confidence levels (i.e., 0.05 level). The comparison between different superscript letter meaning that the difference is statistically significant (Z-Tests).

## Data Availability

Data is available upon request.

## Supplementary Materials

The following supporting information can be downloaded at: https://www.mdpi.com/article/10.3390/ijerph192417040/s1, Table S1: Questionnaire, Table S2: Interpretation of items based on Bandura’s moral disengagement theory.
