# Supplementary material for: Did They Deserve It? Adolescents’ Perception of Online Harassment in a Real-Case Scenario
Source: Int J Environ Res Public Health. 2022 Dec 19;19(24):17040. doi: 10.3390/ijerph192417040 (PMC9778851; doi:10.3390/ijerph192417040)
Supplement: Supplementary file 1 [file ijerph-19-17040-s001.zip › ijerph-2070053-supplementary.pdf]

Supplementary Materials

Table S1. Questionnaire

Gender: ☐ Male ☐ Female

Age: _____

Attended School Class: ☐ Middle School ☐ High School Biennium ☐ High School Triennium

Dear Student,

We are conducting research on adolescents' behaviors on Social Networks. Your participation, which will be completely anonymous, may help us in the data collection for our research. We kindly ask you to answer all of the question with your full honesty.

We will start by asking you with some information about your Social Network use: mark one answer only (unless otherwise specified). There are no correct or wrong answers, so feel free to answer according to your personal opinion.

1. **Why do you use these social networks?** (*You can check multiple answers*)
   - ☐ Socializing
   - ☐ Curiosity
   - ☐ Show more sides of me
   - ☐ Flirting
   - ☐ Find information
2. **What kind of material do you share most?** (*You can check multiple answers*)
   - ☐ Photos
   - ☐ Videos
   - ☐ Messages
   - ☐ Tweets
   - ☐ News
   - ☐ Others
3. **Which social networks do you use the most?** (*You can check multiple answers)*
   - ☐ Facebook
   - ☐ Instagram
   - ☐ WhatsApp
   - ☐ Twitter
   - ☐ Other
4. **Through which devices?** (*You can check multiple answers*)
   - ☐ Smartphone
   - ☐ Shared Laptop
   - ☐ Personal Laptop
   - ☐ Tablet
   - ☐ Others
5. **Who do you think the material you share is accessible to?**

- ☐ Everyone
- ☐ Recipient
- ☐ My network only
- ☐ Adults
- ☐ Other

6. **How much do you use social networks in a day?**
   - ☐ Never
   - ☐ Almost never
   - ☐ Rarely
   - ☐ Sometimes
   - ☐ Almost always
   - ☐ Always
7. **How widespread do you think the material you share is?**
   - ☐ Not at all spread
   - ☐ Low spread
   - ☐ Slightly spread
   - ☐ Moderately spread
   - ☐ Very spread
   - ☐ Extremely spread

Now, you will find an event that may occur to anyone. We ask you to read it and reflect on the situation and its characters before answering the following questions. Again, there are no correct or wrong answers, so feel free to answer according your own opinion.

> *"Fabio and Edoardo, both 16 years old, are deemed responsible for destroying Jessica's reputation by spreading a consensual sexual video between Jessica and Edoardo.*
> *Francesco (16 years old) and Ludovica (17 years old) take action to defend Jessica, by insulting them, creating photomontages with heavy sexual allusions against them, threatening them with death, and intimidating them on social networks."*

	**Not at all**	**Low**	**Slightly**	**Moderately**	**Very**	**Extremely**
**1. How realistic do you think the story is?**	☐	☐	☐	☐	☐	☐
**2. How seriously do you think the story is?**	☐	☐	☐	☐	☐	☐
**3. Could this ever happen in the area where you live?**	☐	☐	☐	☐	☐	☐
**4. Do you think similar events could happen to someone you know?**	☐	☐	☐	☐	☐	☐

Fabio and Edoardo

	Not at all	Low	Slightly	Moderately	Very	Extremely
**5. In your opinion, did Fabio and Edoardo foreseen the consequences of their behaviour?**	☐	☐	☐	☐	☐	☐
**6. Would you ever do what Fabio and Edoardo did?**	☐	☐	☐	☐	☐	☐
**7. In your opinion, what Fabio and Edoardo underwent from Francesco and Ludovica is right?**	☐	☐	☐	☐	☐	☐
**8. Do you think Fabio and Edoardo broke any laws?**	☐	☐	☐	☐	☐	☐

Jessica

	Not at all	Low	Slightly	Moderately	Very	Extremely
**9. In your opinion, Jessica foreseen the consequences of her behaviour?**	☐	☐	☐	☐	☐	☐
**10. Would you ever do what Jessica did?**	☐	☐	☐	☐	☐	☐
**11. Do you think Jessica broke any laws?**	☐	☐	☐	☐	☐	☐

Francesco and Ludovica

	Not at all	Low	Slightly	Moderately	Very	Extremely
**12. In your opinion, did Francesco and Ludovica foreseen the consequences of their behaviour?**	☐	☐	☐	☐	☐	☐
**13. Would you ever do what Francesco and Ludovica did?**	☐	☐	☐	☐	☐	☐
**14. Francesco and Ludovica reaction against Fabio and Edoardo is justifiable?**	☐	☐	☐	☐	☐	☐
**15. Do you think Francesco and Ludovica broke any laws?**	☐	☐	☐	☐	☐	☐

Table S2. Interpretation of items based on Bandura's moral disengagement theory

	Justifying the behavior	*Shifting responsibility*	*Minimizing the harm caused*	*Shifting the causal focus to the victim*
**5. Did Fabio and Edoardo foreseen the consequences of their behaviour?**	Lower scores may imply a justification for the behavior, such as "a joke".	-	Lower scores may imply the underestimation of the severity of potential consequences of non-consensual sharing of intimate images as unlikely or unharmful.	Higher scores may imply a justification for victim-blaming in cyberbullying.
**6. Would you ever do what Fabio and Edoardo did?**	-	Higher scores may indicate a diffusion of responsibility since many people do it.	Higher scores may imply the underestimation of the severity of potential consequences of non-consensual sharing of intimate images as unlikely or unharmful.	Higher scores may imply a justification for victim-blaming in non-consensual sharing after the consensual videotaping.
**7. What Fabio and Edoardo underwent from Francesco and Ludovica is right?**	Higher scores may justify cyberbullying behavior when compared with the non-consensual sharing of intimate images	-	Higher scores may imply the underestimation of the severity of potential consequences cyberbullying as unlikely or unharmful.	Higher scores may indicate that after the non-consensual sharing of intimate images, the cyberbullying is deserved
**9. Jessica foreseen the consequences of her behaviour?**	-	-	Lower scores may imply the underestimation of the severity of potential consequences of consensual videotaping as unlikely or unharmful.	Higher scores may imply victim blaming after consenting videotaping
**10. Would you ever do what Jessica did?**	-	Higher scores may indicate a diffusion of responsibility since "everyone does it"	-	Lower scores may imply that consensual videotaping is strictly linked to non-consensual sharing, and, therefore, to victim blaming.
**12. Did Francesco and Ludovica foreseen the consequences of their behaviour?**	Higher scores may indicate a justification of the behavior by invoking moral values (e.g., justice) or deeming cyberbullying as less serious than the non-consensual sharing of intimate images	-	Lower scores may imply the underestimation of the severity of potential consequences cyberbullying as unlikely or unharmful.	-

**13. Would you ever do what Francesco and Ludovica did?**	Higher scores may indicate a justification of the behavior by invoking moral values (e.g., justice)	Higher scores may indicate a diffusion of responsibility since "everyone would do it"	-	Higher scores may justify the cyberbullying behavior through the victim blaming.
**14. Francesco and Ludovica reaction against Fabio and Edoardo is justifiable?**	Higher scores may imply a consideration of cyberbullying as less serious than the non-consensual sharing of intimate images	-	Higher scores may imply the underestimation of the severity of potential consequences cyberbullying as unlikely or unharmful.	Higher scores may justify the cyberbullying behavior through the victim blaming.

Note. *a. Items related to the event's features (items 1, 2, 3, and 4) represent a measure of the credibility and closeness of the scenario, while items related to the violation of the law (items 8, 11, and 15) allow measuring awareness about cybercrime; therefore, these 7 items were not interpreted from the perspective of Bandura's moral disengagement theory. b. cybercrimes were the following: Production and possession of child pornography (Jessica, Fabio, and Edoardo); non-consensual sharing of intimate images and distribution of child pornography (Fabio and Edoardo); cyberbullying (Ludovica and Francesco).*
